# Supplementary material for: Critical Influence of Water on the Polymorphism of 1,3-Dimethylurea and Other Heterogeneous Equilibria
Source: Molecules. 2023 Oct 12;28(20):7061. doi: 10.3390/molecules28207061 (PMC10609064; doi:10.3390/molecules28207061)
Supplement: Supplementary file 1 [file molecules-28-07061-s001.zip › molecules-2618069-supplementary.docx]

SUPPORTING INFORMATION

Critical influence of water on the polymorphism of 1,3-dimethylurea and other heterogeneous equilibria.

Grace Baaklini ^1^, Manon Schindler ^1^, Lina Yuan ^1^, Clément De Saint Jores ^1^, Morgane Sanselme ^1^,
Nicolas Couvrat ^1^, Simon Clevers ^1^, Philippe Négrier ^2^, Denise Mondieig ^2^, Valérie Dupray ^1^,
Yohann Cartigny ^1^, Gabin Gbabode ^1,^* and Gerard Coquerel ^1,^*

^1^ Laboratoire Sciences et Méthodes Séparatives UR3233, Université Rouen Normandie,
Normandie Université, F-76000 Rouen, France; grace.baaklini@dsm-firmenich.com (G.B.);
manon.schindler@servier.com (M.S.); lina.yuan@novartis.com (L.Y.);
clement.de-saint-jores@univ-orleans.fr (C.D.S.J.); morgane.sanselme@univ-rouen.fr (M.S.);
nicolas.couvrat@univ-rouen.fr (N.C.); simon.clevers@gmail.com (S.C.); valerie.dupray@univ-rouen.fr (V.D.);
yohann.cartigny@univ-rouen.fr (Y.C.)

^2^ LOMA, UMR 5798, Université Bordeaux, 351 Cours de la Libération, F-33400 Talence, France;
philippe.negrier@u-bordeaux.fr (P.N.); denise.mondieig@u-bordeaux.fr (D.M.)

* Correspondence: gabin.gbabode@univ-rouen.fr (G.G.); gerard.coquerel@univ-rouen.fr (G.C.)

Table of contents

S1. Reproducibility of the polymorphic transition of DMU

Figure S1. DSC curves of a DMU sample obtained for two subsequent heating-cooling cycles performed at a scanning rate of 5 °C / min.

S2. Estimation of the amount of water in the reactor containing 7 g of DMU in 80 mL of n-heptane

S3. Additional data related to the identification and structural characterization of the DMU monohydrate and additional details on its crystal packing

Figure S2. TR-X-ray powder diffraction patterns of the DMU mohohydrate.

Figure S3. XRPD patterns of DMU monohydrate measured after up to 4 heating-cooling cycles between -25 °C and -10 °C.

Figures S4 – S6. Projections of the C2/c crystal structure of the DMU monohydrate along different directions with particular emphasis on the particular molecular chains formed.

Figure S7. Projections of the alternative Iba2 crystal structure of DMU monohydrate.

Table S1 Crystallographic data determined for DMU monohydrate *C*2/c and *I*ba2 crystal structure solutions.

S4. Additional data for the construction of the DMU - water binary phase diagram

Figure S8. Tammann graph of the peritectic invariant at 8 °C

Figure S9. Calibration curve of the refractive index of different DMU-water mixtures as a function of the mass percentage of water (measured at room temperature)

Table S2. Solubility of DMU (expressed in molar fraction of water) obtained by refractometry at different temperatures

Table S3. Liquidus and metastable eutectic invariant temperatures determined by TR-SHG for several DMU-water compositions

S5. Purity determination of commercial and recrystallized DMU

Figure S10. HPLC chromatogram of commercial DMU

Table S4. Chromatographic data of commercial DMU

Figure S11. HPLC chromatogram of recrystallized DMU

Table S5. Chromatographic data of recrystallized DMU

Figure S12. DSC thermograms of commercial and recrystallized DMU.

Figure S13. DSC thermograms of DMU commercial and recrystallized samples enriched with water

S6. Additional experimental details

Figure S14. Schematic representation of In-situX^®^ setup

S1. Reproducibility of the polymorphic transition of DMU

DSC measurements were carried out using a Netzsch DSC 204 F1 apparatus for which cooling is provided by automated liquid nitrogen flux. Each DSC run was realised with a few mg of powdered DMU sample (± 0.05 mg) in aluminum pans with pierced lids. The atmosphere for the analyses was regulated by nitrogen gas flux (40 mL / min). Heating-cooling cycles were performed between -130°C and 90°C at a scanning rate of 5 °C / min. It appears that crystallization of form II of DMU occurs at low temperature (below -60°C). Upon heating, the form II to form I transition occurs at a temperature of around 42 °C reproducibly in the two heating scans shown in Figure S1. Hence, for a given sample (with a fixed water content –not measured-), no variation of the temperature of transition is observed through subsequent heating-cooling cycles at the same scanning rate, thus disregarding any kinetic effects. It appears that new solid forms of DMU have been revealed at very low temperature by temperature dependent X-ray diffraction (ongoing study) that might actually be responsible of the multiple exothermic peaks observed upon cooling at very low temperature (less than -80°C) and of the small endothermic peaks observed at around -120°C and 10°C upon the first and second heating cycles.


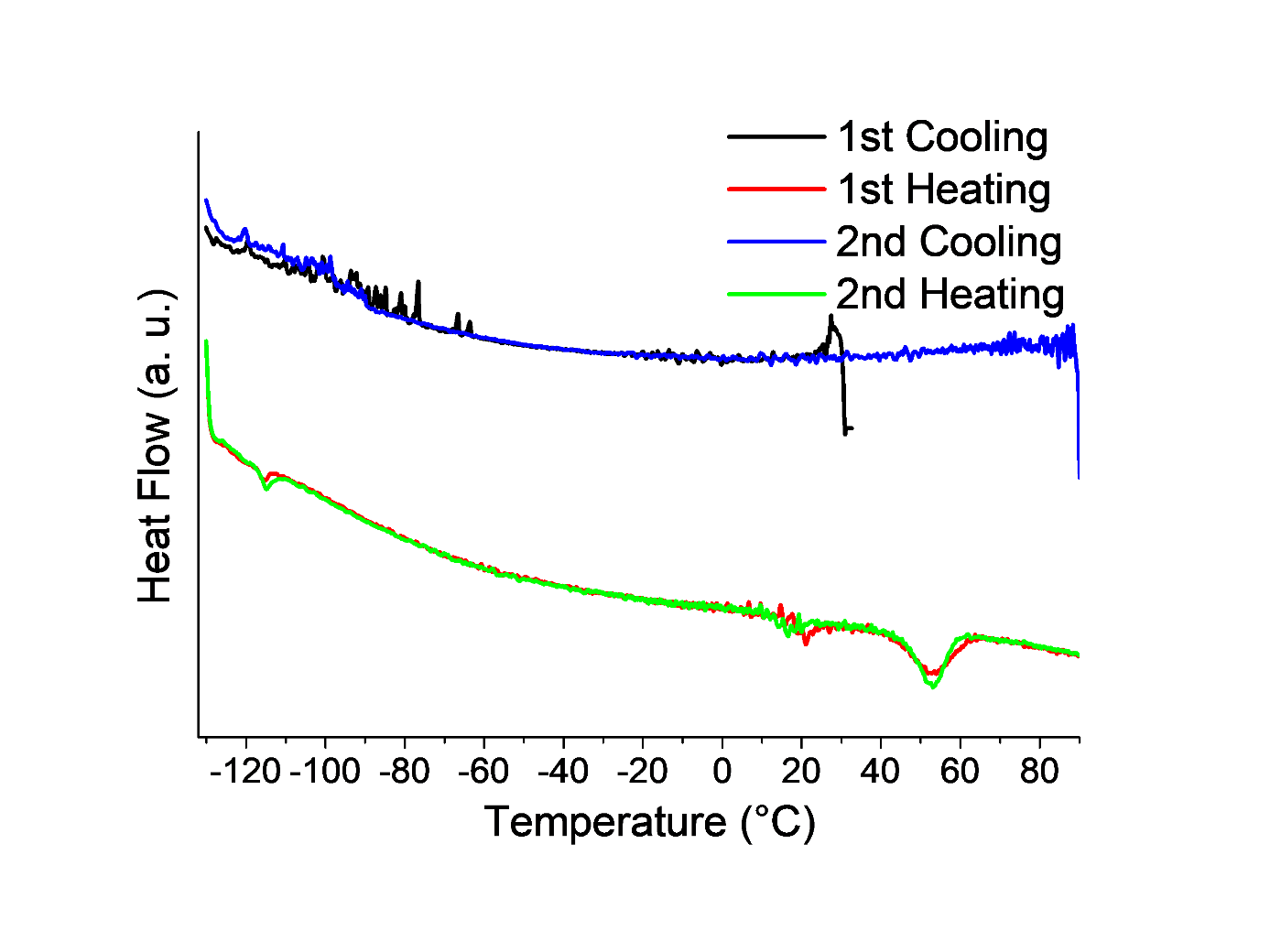


Figure S1. DSC curves of a DMU sample obtained for two subsequent heating-cooling cycles performed at a scanning rate of 5 °C / min.

S2. Estimation of the amount of water in the reactor containing 7 g of DMU in 80 mL of n-heptane

n-heptane dried with molecular sieve

The amount of water in the heptane dried with molecular sieve was found to be 40 ppm by Karl Fischer titration.

Volume heptane = 80 mL; [ρ](https://fr.wikipedia.org/wiki/Rh%C3%B4) = 0.6795 g / mL ; mass heptane = [ρ](https://fr.wikipedia.org/wiki/Rh%C3%B4) × V = 0.6795 x 80 = 54.36 g

(54.36 × 40) / 7 = 310 ppm which corresponds to 0.03 % of water in mass fraction, thus corresponding to 0.15 % in molar fraction (M (H_2_O) = 18 g / mol and M (DMU) = 88.13 g / mol).

n-heptane saturated with water

The amount of water in the n-heptane saturated in water was found to be 98 ppm by Karl Fischer titration. The same formula applied above gives a value of 775 ppm of water inside the reactor which corresponds to 0.39 % of water in molar fraction.

S3. Additional data and related to the identification and structural characterization of the DMU monohydrate and additional details on its crystal packing

Figure S2. TR-X-ray diffraction patterns of the DMU mohohydrate. At -25 °C the characteristic peaks of ice are indicated by arrows. At 11 °C, above the peritectic transition, Form II crystallizes as shown by the presence of its characteristic peaks.


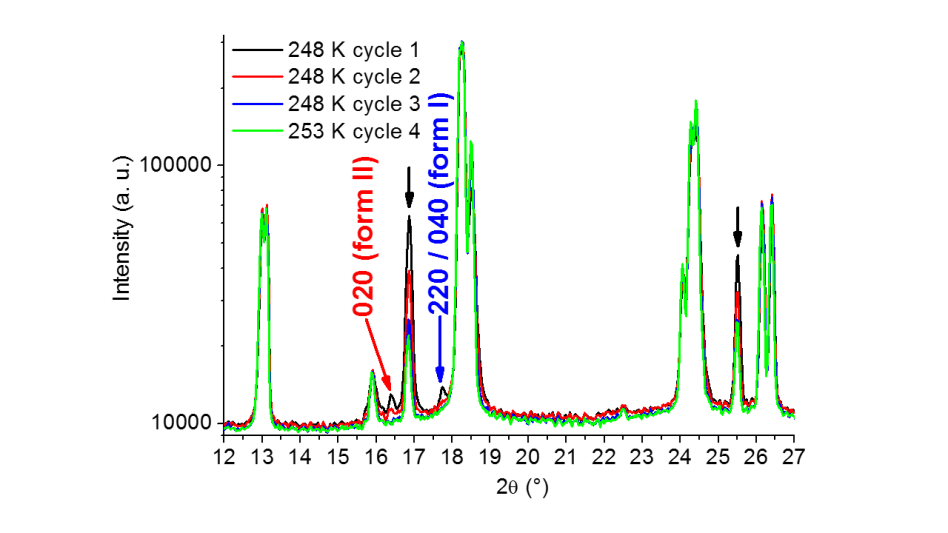


Figure S3. XRPD patterns of DMU monohydrate represented in the [12°-27°] 2θ range measured at -25 °C (black, red and blue diagrams) or -20 °C (green diagram) after up to 4 heating-cooling cycles between -25 °C and -10 °C. Peaks pointed by blue and red arrows are assigned to anhydrous forms I and II, respectively. After 4 cycles, those peaks are no longer visible. To the contrary, the two peaks at 16.9° and 25.5° in 2θ which could not be assigned to a known form (including that of DMU monohydrate) remain after 4 cycles even if their intensities have sensibly decreased from the first to the fourth cycle.

Detailed analysis of the crystal packing of the DMU monohydrate

A finer analysis of the crystal structure of DMU monohydrate reveals that water molecules arrange into infinite channels oriented parallel to the *b* axis as shown in Figure S4 which displays projections of the crystal structure of DMU monohydrate where DMU molecules have been removed for clarity. In Figure S4b, two color codes (red and white; black) are used for water molecules to differentiate homologues generated by the two symmetry independent water molecules of the asymmetric unit. Each channel is separated from its neighbors by a distance of approximately 0.5 × *a* (= 9.6 Å) along *a* axis and 0.5 × *c* (= 6.7 Å) along *c* axis (Figure S8a). Furthermore, along a given channel, each water molecule is almost equidistant from the subsequent or preceding one by a distance of approximately 0.25 × *b* (= 3.5 Å).

These spare spaces are then filled by DMU molecules which then locate in between every two water molecules along the channel axis and in between neighboring channels, then forming infinite chains of alternate DMU and water molecules connected by hydrogen bonds (Figures S5 and S6). Due to the presence of two DMU molecules with different orientations in the asymmetric unit, two types of chains with orthogonal orientations arise: chains A which are oriented parallel to *c* axis and chains B oriented parallel to *a** reciprocal axis (that is, perpendicular to both *b* and *c* axes), all DMU molecules within a given chain keeping the same orientation. Along the water channels, those chains alternate in an ABAB… fashion (Figure S5b) from one water – water interstice to the other or more precisely in an A_1_B_1_A_2_B_2_A_1_B_1_A_2_B_2_… fashion since for every two closest neighboring chains of the same kind, DMU molecules point to opposite direction in an antiparallel way, then giving rise to A_1_ and A_2_ or B_1_ and B_2_ chains (see Figures S5b and S6a). Noticeably, within a given chain of any type, subsequent DMU molecules are not coplanar but rather tilted of an angle of around 66° from one another (Figure S5b).


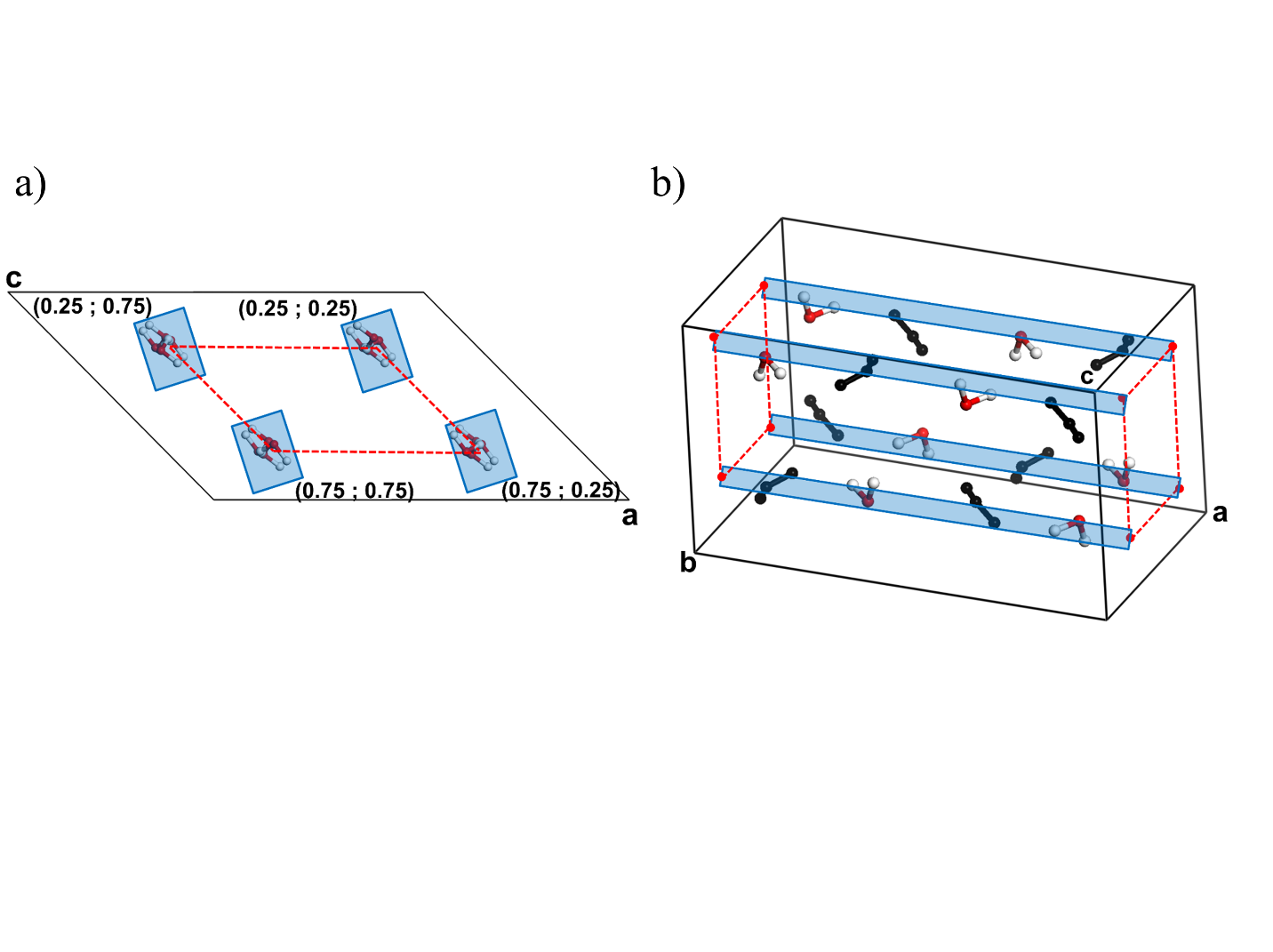


Figure S4. a) Projection in the (*a*, *c*) plane and b) perspective view of the crystal structure of DMU monohydrate where DMU molecules have been removed to emphasize the arrangement of water molecules in channels (highlighted in blue) parallel to *b* axis. In a) numbers between brackets indicate the approximate fractional coordinates in the (*a*, *c*) plane of the 4 water channels present in the unit cell. In b) molecules of the same colour are symmetrically related while they are not for water molecules of different colours (Z’ = 2).


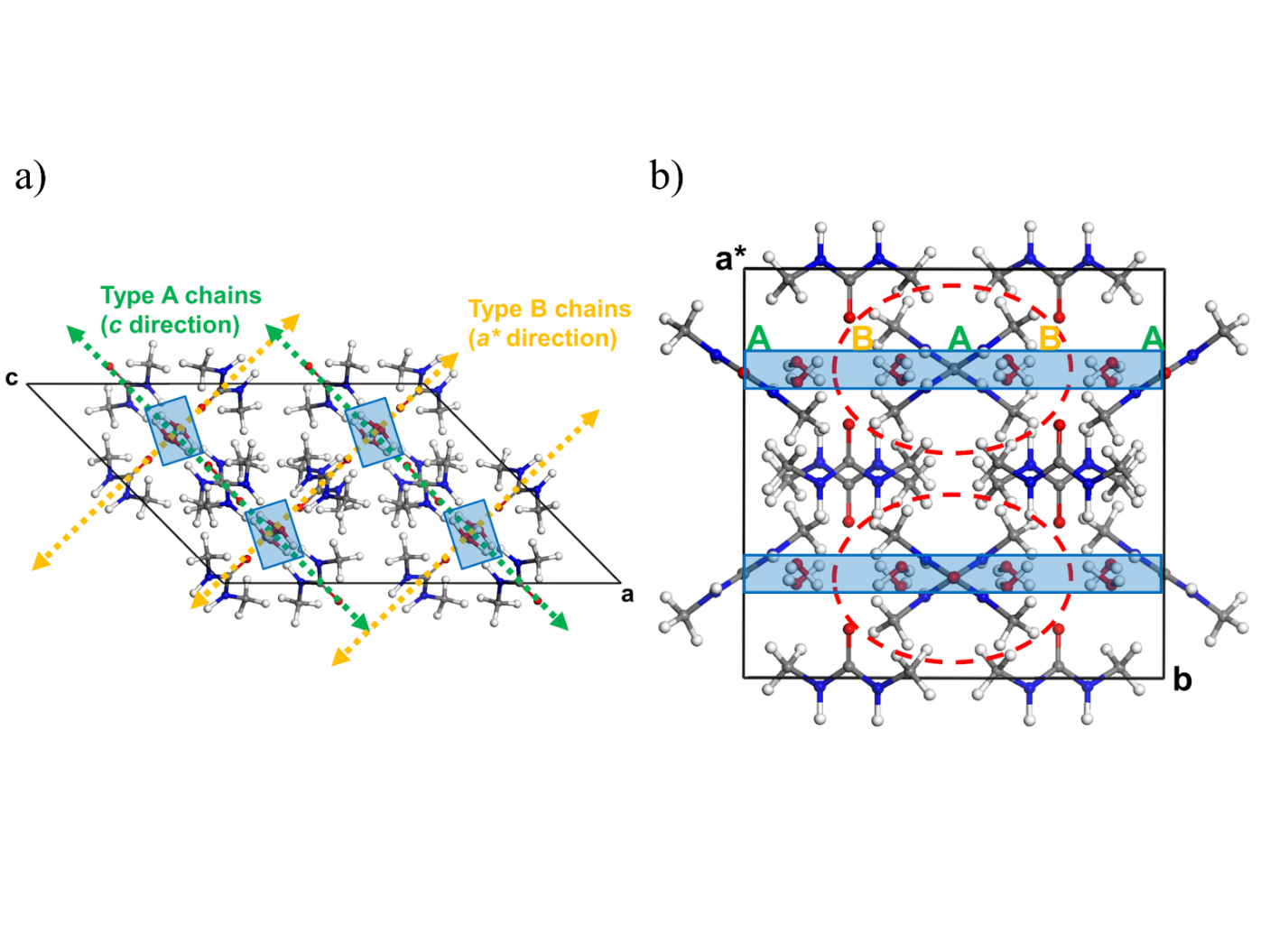


Figure S5. Projections in the a) (*a*, *c*) plane and b) (*a**, *b*) plane of the crystal structure of DMU monohydrate. Water channels are highlighted in blue. Hydrogen bonds between water and DMU molecules are not shown for clarity. In a) the two types of molecular chains are emphasized by respectively green (type A) and orange (type B) dashed double arrows. In b) the ABAB… alternation along the water channel is represented by the corresponding letters in the same colour code as used in a). Dashed red ellipses pinpoint DMU molecules of the same chain (type A here) for which the tilt angle between the respective molecular planes of two neighbouring molecules is distinctively visible.


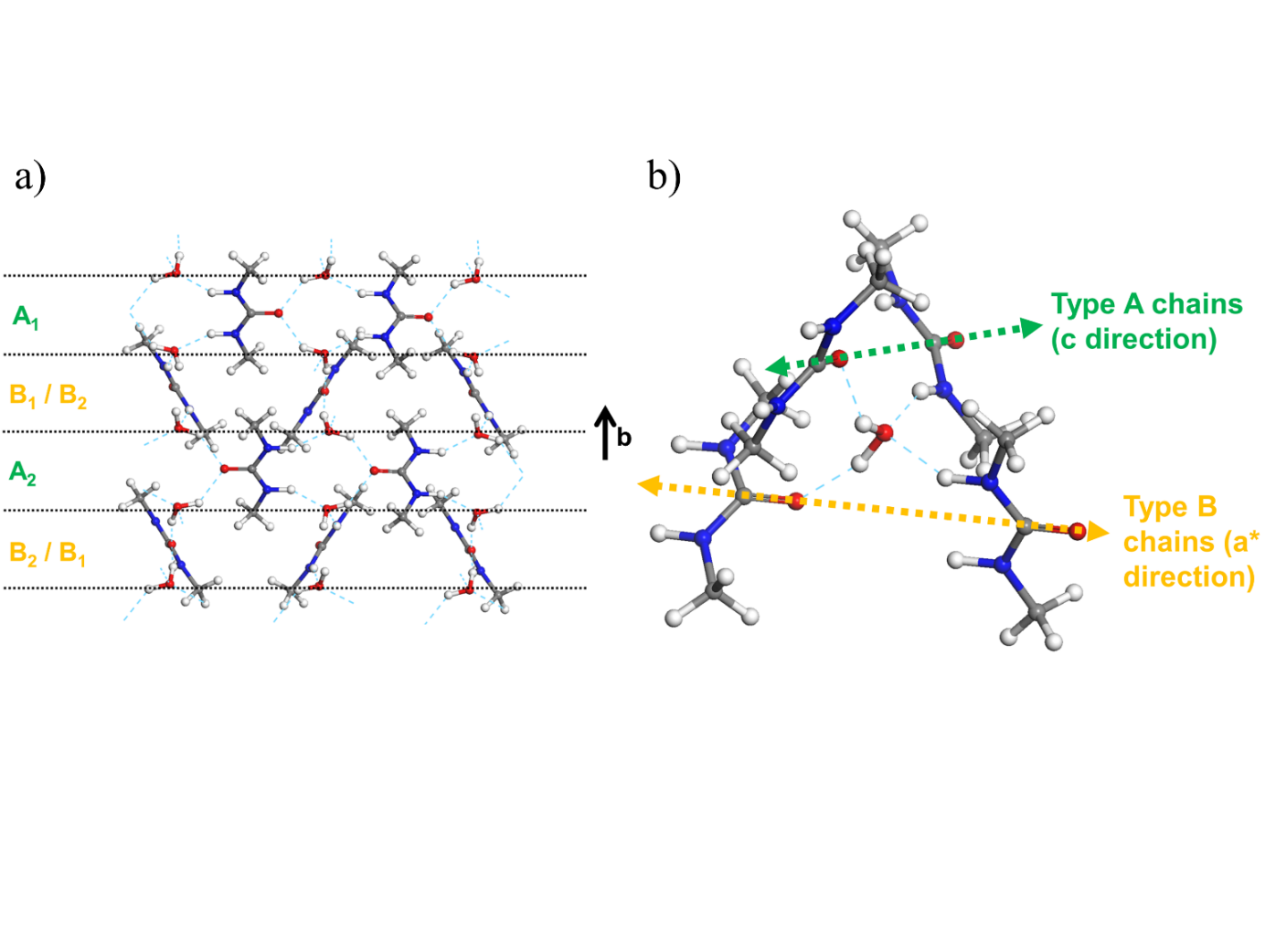


Figure S6. Hydrogen bonding network in the crystal structure of DMU monohydrate. a) View of the crystal structure with the water channels oriented vertically. Alternations between A and B chains (for any two subsequent water – water interstices) and between A_1_ and A_2_ or B_1_ and B_2_ chains (for every three water – water interstices) are well visible. b) Representation of the 4 hydrogen bonds in which every water molecule is involved, with two DMU molecules of A chains and two DMU molecules of B chains.

Lastly, it has to be mentioned that the crystal structure of DMU monohydrate could be solved with a slightly poorer agreement factor in an orthorhombic *I*ba2 crystal lattice. The unit cell parameters were very similar and the crystal packing was essentially the same (Table S1 and Figure S7). However, Second Harmonic Generation (SHG) measurements performed on DMU monohydrate powder clearly indicated that its crystal structure had to be centrosymmetric since no SHG signal was detected. The centrosymmetric *C*2/c crystal structure was thus kept and the non-centrosymmetric *I*ba2 crystal structure discarded.


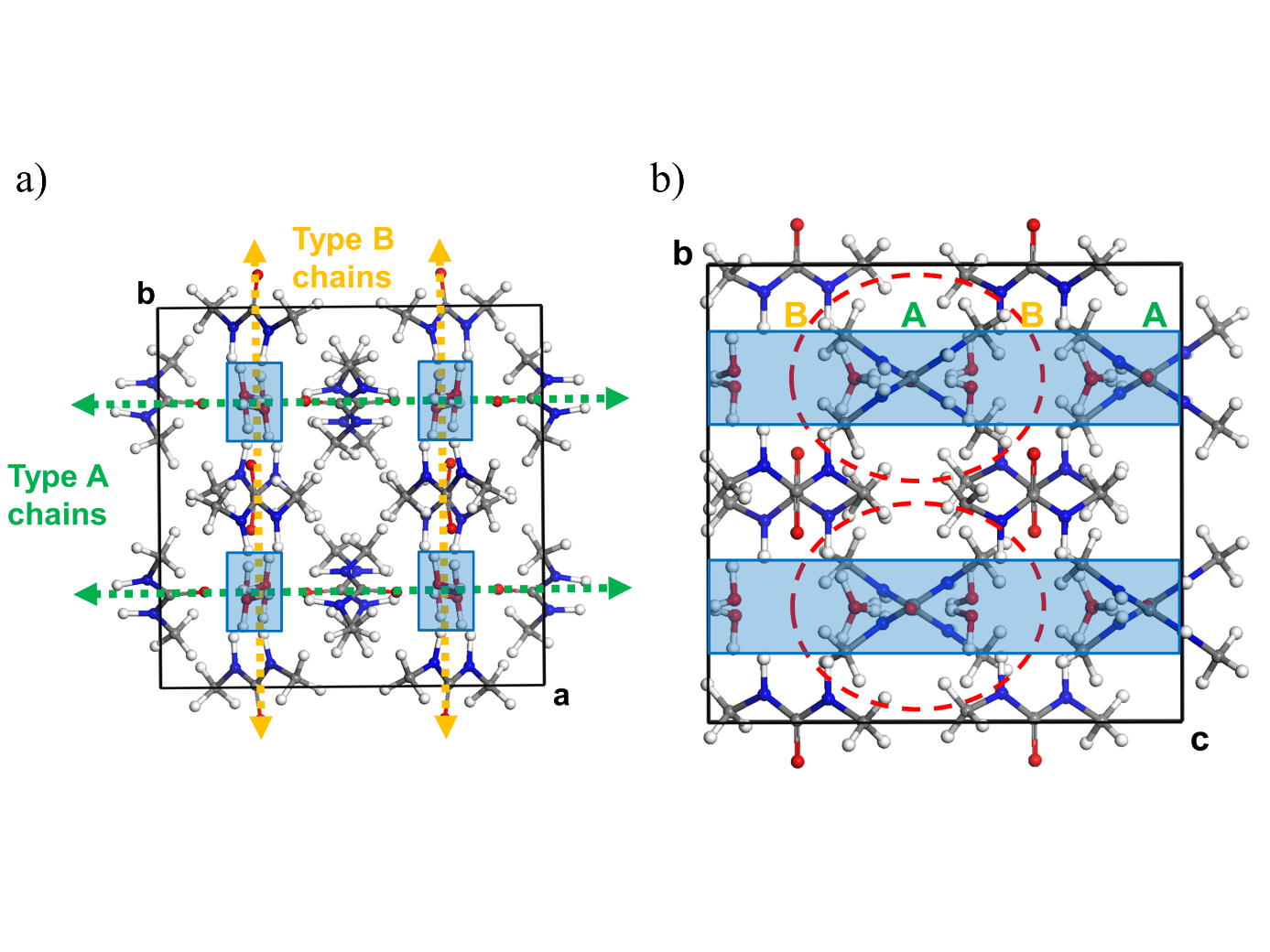


Figure S7. Projections in the a) (*a*, *b*) plane and b) (*b*, *c*) plane of the *I*ba2 crystal structure of DMU monohydrate. Water channels are highlighted in blue. Hydrogen bonds between water and DMU molecules are not shown for clarity. In a) the two types of molecular chains are emphasized by respectively green (type A) and orange (type B) dashed double arrows. In b) the ABAB… alternation along the water channel is represented by the corresponding letters in the same color code used in a). Dashed red ellipses pinpoint DMU molecules of the same chain (type A here) for which the tilt angle between the respective molecular planes of two neighbouring molecules is distinctively visible.

Table S1 Crystallographic data determined for DMU monohydrate *C*2/c and *I*ba2 crystal structure solutions.

| Form | DMU monohydrate | |
| --- | --- | --- |
| Temperature (K) | 253 | 253 |
| Crystal system | Monoclinic | Orthorhombic |
| Space group | *C*2/c | *I*ba2 |
| Rwp | 0.048 | 0.058 |
| *a* (Å) | 19.1479 (8) | 13.6230 (5) |
| *b* (Å) | 13.9557 (5) | 13.4920 (5) |
| *c* (Å) | 13.4926 (4) | 13.9571 (6) |
| *β* (°) | 134.644 (2) | 90 |
| Z | 16 | 16 |
| Z’ | 2 | 2 |
| Volume (Å^3^) | 2565 (1) | 2565 (1) |
| Density (g/cm^3^) | 1.099 (1) | 1.099 (1) |

S4. Additional data for the construction of the DMU - water binary phase diagram

Table S2. Solubility of DMU (expressed in molar fraction of water) obtained by refractometry at different temperatures

| Molar fraction of water (%) | Temperature (°C) |
| --- | --- |
| 62.47 | 16 |
| 62.59 | 15 |
| 62.47 | 14 |
| 63.6 | 12 |
| 64.65 | 10 |
| 63.86 | 10 |
| 65.08 | 8 |
| 67.37 | 5 |
| 67.3 | 0 |
| 69.76 | -5 |
| 71.02 | -10 |
| 72.11 | -15 |


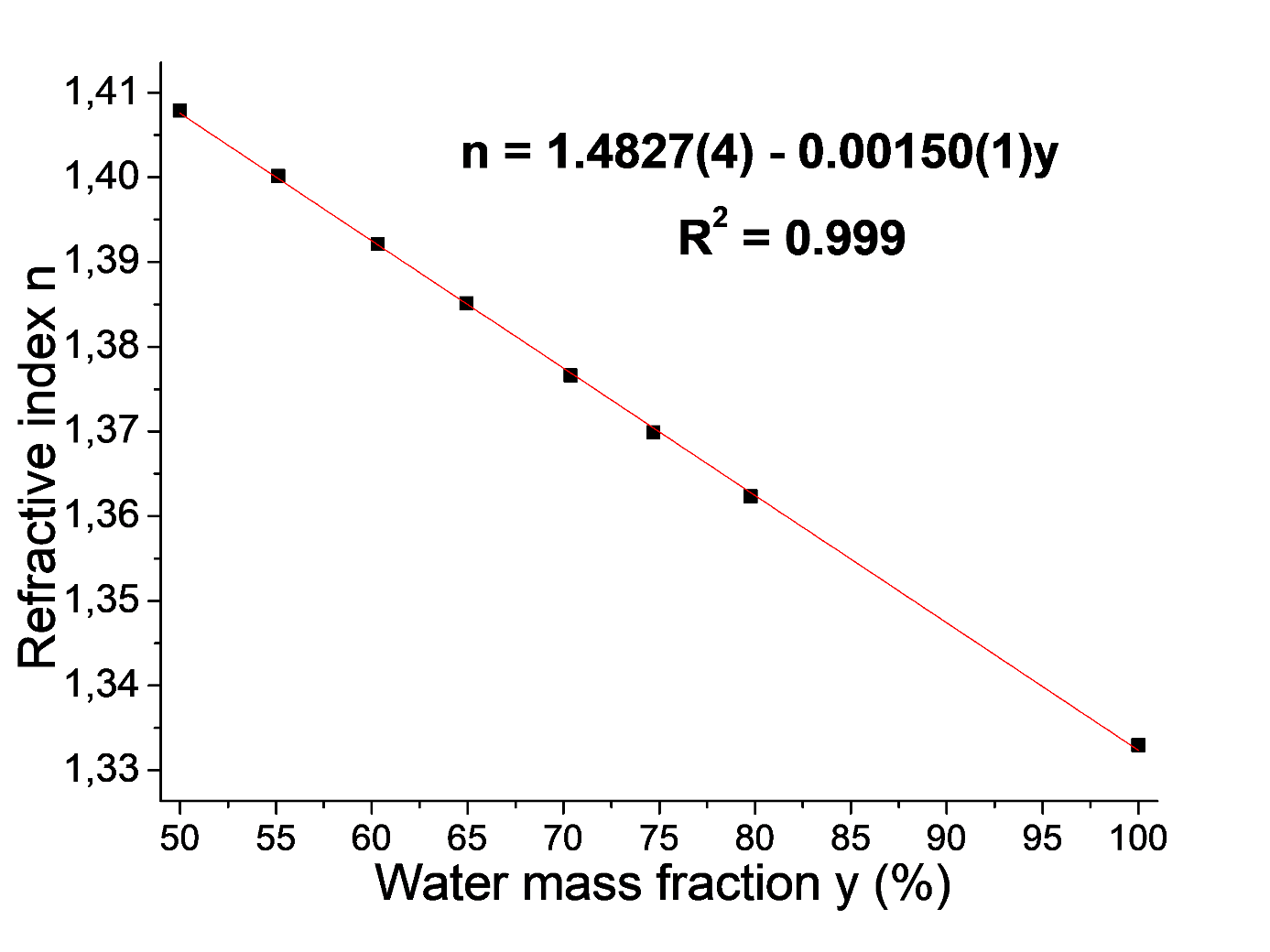


Figure S8. Calibration curve of the refractive index of different DMU-water mixtures as a function of the mass percentage of water (measured at room temperature).


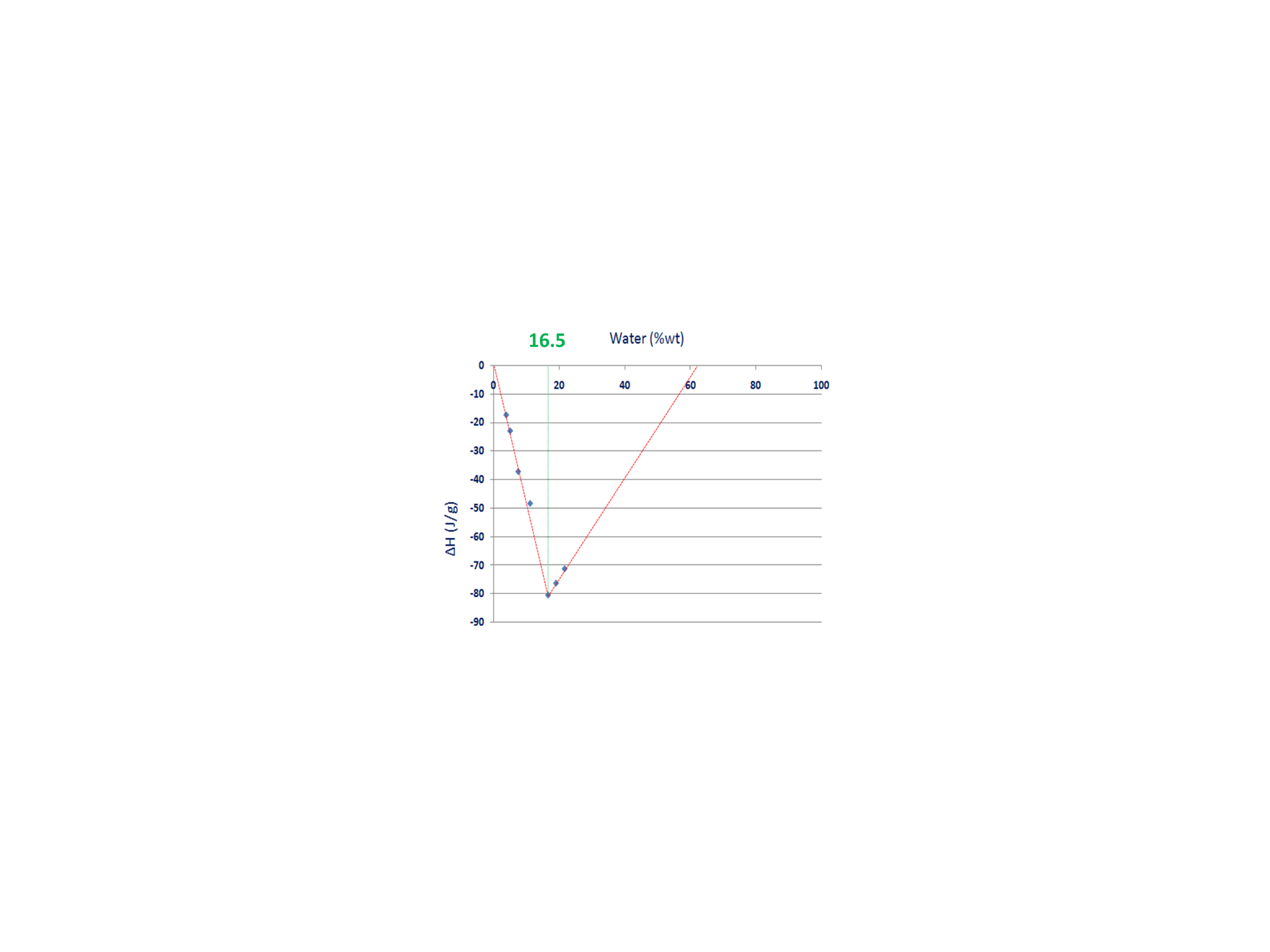


Figure S9. Tammann graph of the peritectic invariant at 8 °C: the heats of fusion of peritectic invariant at 8 °C (determined by DSC) were plotted versus water composition in mass percentage; the highest heat of fusion of the peritectic was found at 16.5% weight percent in water corresponding to a molar composition of 49.2 % in water fairly close to 50% which further confirms that the hydrate stoichiometry is DMU:water (1:1).

Table S3. Liquidus and metastable eutectic invariant temperatures determined by TR-SHG for several DMU-water compositions (in molar fraction).

| Molar composition  in water (%) | Liquidus temperature (°C) | Metastable eutectic temperature (°C) |
| --- | --- | --- |
| 60 | 23.8 | -37.5 |
| 62 | 21.3 | -37.5 |
| 65 | 10 | -37.5 |
| 68 | -12.5 | -37.5 |
| 70 | -17.5 | -37.5 |
| 71 |  | -37.5 |
| 75 |  | -37.5 |

S5. Purity determination of commercial and recrystallized DMU

The commercial DMU was recrystallized from ethanol upon two cycles. HPLC and DSC analyses showed that the recrystallized DMU is purer than the commercial DMU.

***HPLC analyses of commercial and recrystallized DMU***

The chromatographic measurements were performed with a liquid chromatograph from Thermo-Fisher-Scientific, which consisted of a P680 pump, an injection valve (ASI-100 automated sample injector) equipped with a 10-µL injection loop, and a model UVD 340U diode array detector (DAD). The column type is Thermo Accucore AQ 100 mm x 4.6 mm x 3 µm. The column temperature was controlled at 20 °C with a TCC-100 thermostated column compartment. The data were collected in a computer using the Chromeleon 6.80 software. The mobile phase was composed only of ultra-purified water at a flow rate of 0.3 mL/min. The DAD scanned wavelength from 200 to 470 nm, and chromatograms were presented with a 205 nm wavelength.

The chromatogram of commercial DMU shows that the retention time of DMU is estimated at 22 minutes (Figure S10). The corresponding relative area is 96.23% (Table S4).

The chromatogram of DMU recrystallized from ethanol upon two cycles shows that the retention time of DMU is also estimated at 22 minutes (Figure S11). The corresponding relative area is 99.88% (Table S5).

Hence, HPLC results confirm that recrystallized DMU is purer than commercial DMU.


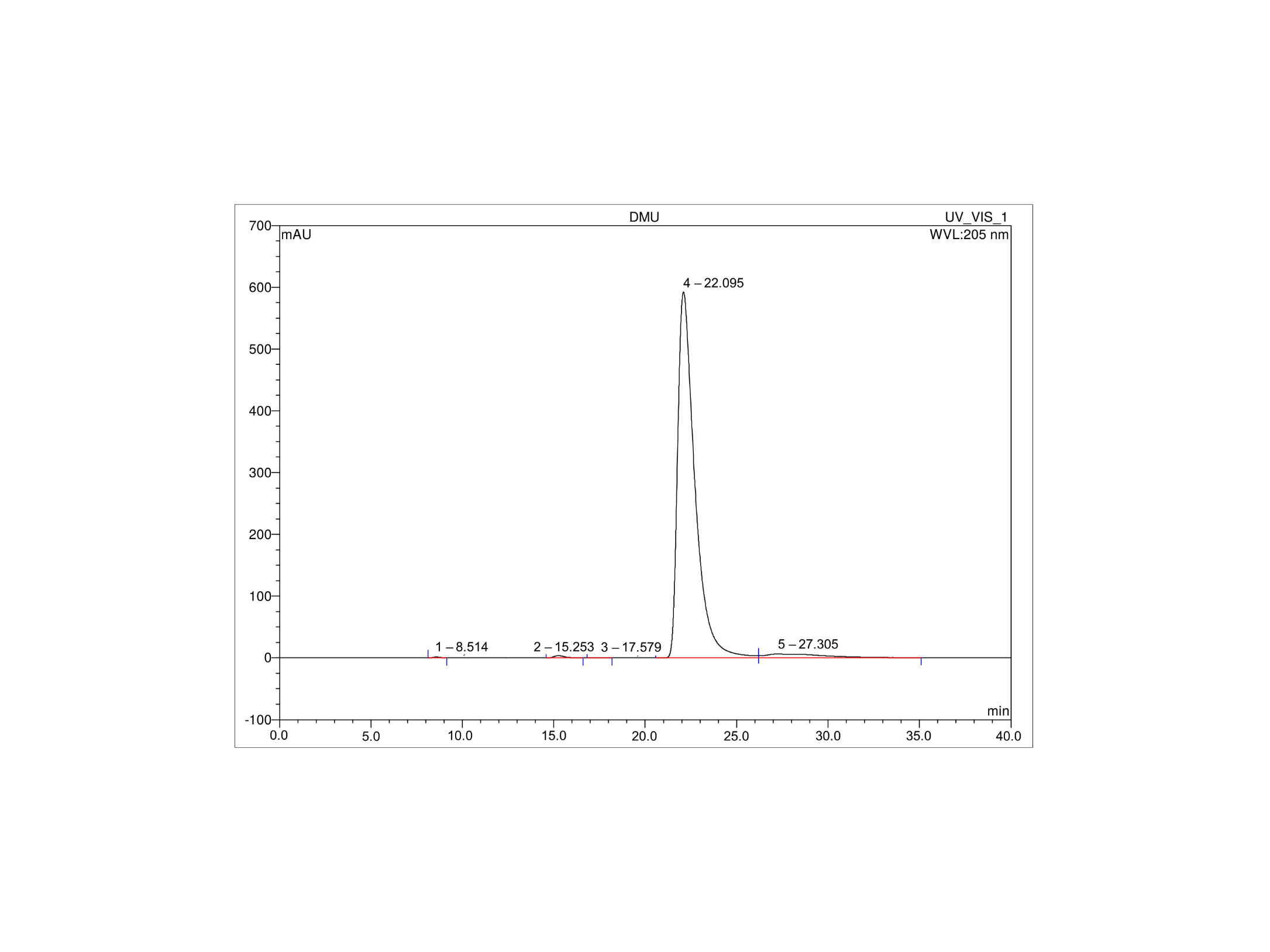


Figure S10. HPLC chromatogram of commercial DMU

| Peak No. | Retention  time (min) | Height  (mAU) | Area  (mAU*min) | Relative Area (%) |
| --- | --- | --- | --- | --- |
| 1 | 8.51 | 1.300 | 0.554 | 0.08 |
| 2 | 15.25 | 3.544 | 2.218 | 0.33 |
| 3 | 17.58 | 0.343 | 0.259 | 0.04 |
| 4 | 22.09 | 592.357 | 641.809 | 96.31 |
| 5 | 27.30 | 6.147 | 22.136 | 3.32 |
| Total: |  | 603.691 | 666.976 | 100.00 |

Table S4. Chromatographic data of commercial DMU


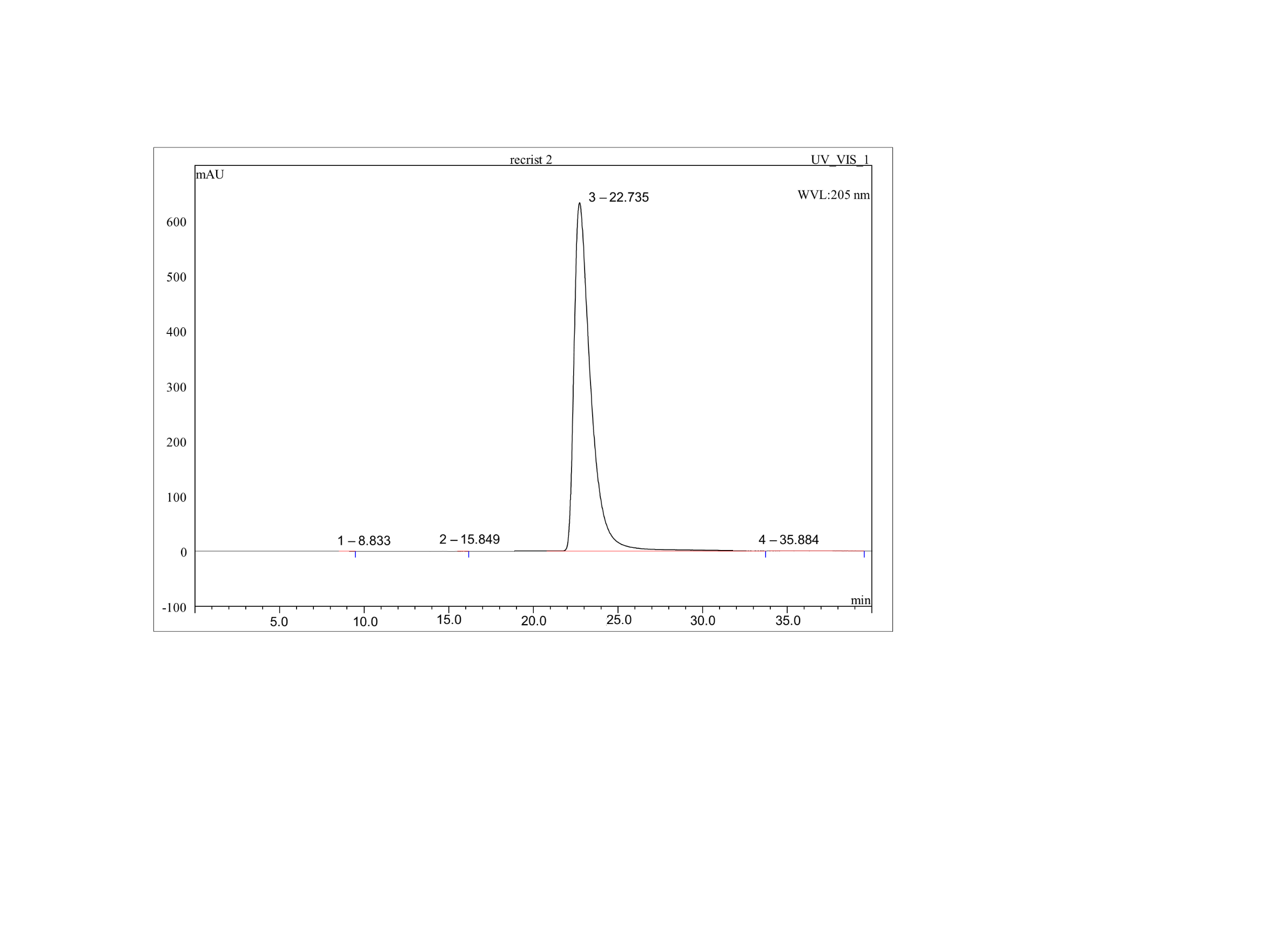


Figure S11. HPLC chromatogram of recrystallized DMU

Table S5. Chromatographic data of recrystallized DMU

| Peak No. | Retention  time (min) | Height  (mAU) | Area  (mAU*min) | Relative Area (%) |
| --- | --- | --- | --- | --- |
| 1 | 8.83 | 0.576 | 0.242 | 0.03 |
| 2 | 15.85 | 0,213 | 0.072 | 0.01 |
| 3 | 22.74 | 632.217 | 717.720 | 99.88 |
| 4 | 35.88 | 0.197 | 0.574 | 0.08 |
| Total: |  | 633.204 | 718.608 | 100.00 |

***DSC analyses of commercial and recrystallized DMU***

**Purity Determination by NETSCH®** is a program for determining the molar purity of a material through analysis of the DSC melting peak using the Van’t Hoff equation

$$T_{j}=T_{0}+\frac{RT_{0}^{2}}{\Delta H}x_{2}^{*}\frac{1+Corr}{F_{j}+Corr}$$

T_0_: melting temperature for the pure substance

T_j_: current temperature

$x_{2}^{*}$: mole fraction of impurity

ΔH: molar melting enthalpy of the sample to analyze

F_j_: fraction melted at the current temperature

Corr: Correction parameter

2 mg of commercial DMU and recrystallized DMU were analysed by DSC with the following program: heating from 20 °C to 60 °C at 10 °C/min then heating from 60 °C up to 130 °C with a heating rate of 1 °C/min. This heating program is recommended for the purity determination by NETSCH^®^.

The DSC thermograms of both samples show a difference in the shape of the melting peak and the melting onset value. Indeed, a sharp melting peak is observed for the recrystallized DMU with T_onset_ = 106.7 °C whereas a broader melting peak is noticed for the commercial DMU with T _onset_ = 104.8 °C. This confirms that the recrystallization of DMU from ethanol leads to a purer product (Figure S12).

Figure S12. DSC thermograms of commercial and recrystallized DMU. Heating rate: 1 °C/min

The following average molar purity were found for DMU:

Commercial DMU: 98.8 %

Purity of recrystallized DMU: 99.9%

Thus, purity determination results obtained by DSC with the support of NETSCH® software are in good agreement with HPLC analyses performed on commercial and recrystallized DMU.

***DSC analyses of commercial and recrystallized DMU enriched with water***

DMU commercial and recrystallized samples were enriched with water (45 molar % in water) and analyzed by DSC with a heating rate of 5 °C/min. Figure S13 shows that both samples underwent exactly the same invariant transitions: metastable and stable eutectic followed by a peritectic at 8 °C and the metatectic at 25 °C (see also section 2.3.1 of the manuscript)

DSC findings prove that water is the only impurity that impacts the solid-solid transition through a metatectic equilibrium decreasing the solid-solid transition from 58 °C down to 25 °C.

Figure S13. DSC thermograms of DMU commercial and recrystallized samples enriched with water (45 % in molar fraction). Heating rate: 5 °C/min

S6. Additional experimental details


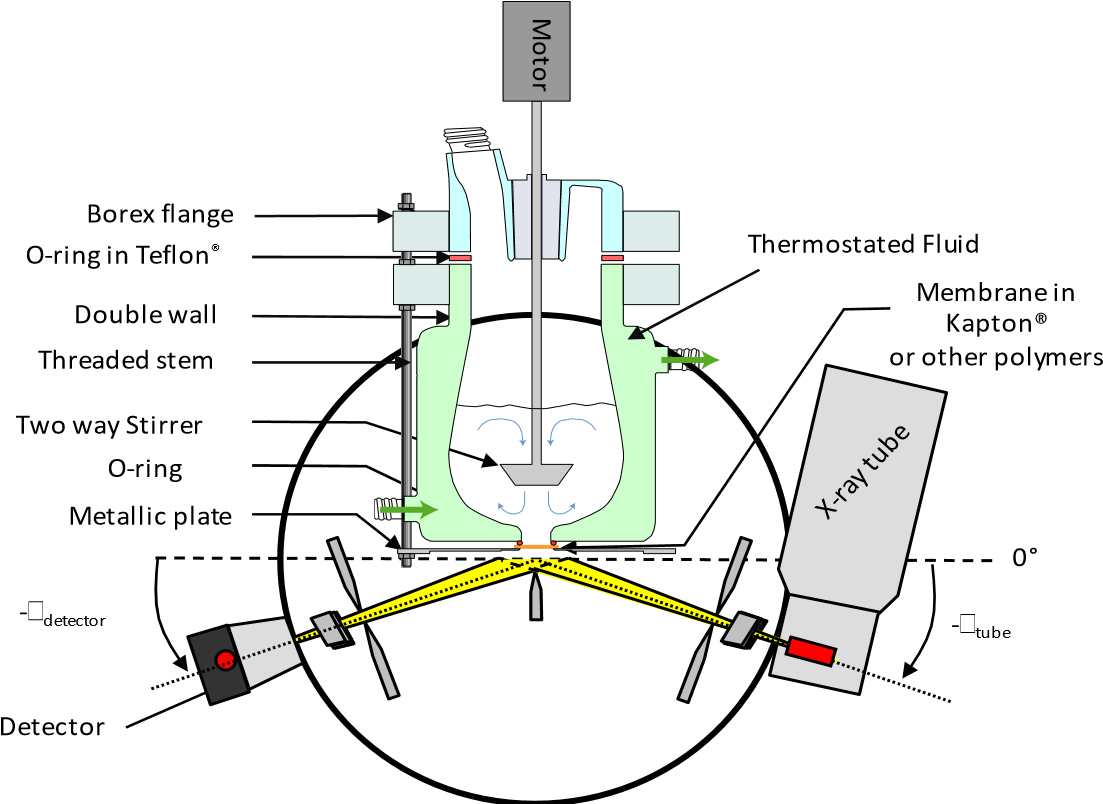


Figure S14. Schematic representation of In-situX^®^ setup

The system is closed and X-ray powder diffraction analyses can be performed without sampling. Thus, the small amount of water which is in the system remains constant throughout the entire experiment.
